# MRI-Based Morphometric Analysis of the Femoral Intercondylar Notch and Its Association With Traumatic Anterior Cruciate Ligament Injury: A Prospective Observational Study in a South Indian Population

**DOI:** 10.7759/cureus.103229

**Published:** 2026-02-08

**Authors:** Prasad Soraganvi, Praveen Kumar Venkataraman, Surendra Babu, Siva Mahesh S

**Affiliations:** 1 Department of Orthopaedics, St. Peter's Medical College, Hospital and Research Institute, Hosur, IND

**Keywords:** acl risk factors, knee morphometry, knee trauma, notch morphology, traumatic knee injury

## Abstract

Introduction: Femoral intercondylar notch morphology has been proposed as an intrinsic anatomical risk factor for anterior cruciate ligament (ACL) injury. Magnetic resonance imaging (MRI) provides a reliable and non-invasive method for assessing notch morphometry and related parameters associated with ACL injury risk.

Methods: A prospective observational study was performed on 108 patients aged 18-45 years who underwent knee MRI following traumatic injury. Participants were divided into a normal ACL group (n = 54) and an ACL tear group (n = 54). Morphometric parameters, including notch width (NW), bicondylar width (BW), NW index (NWI), notch angle, and notch shape, were measured on standardized axial MRI images. Continuous variables were analyzed using independent t-tests, and categorical variables were compared using the chi-square (χ²) test.

Results: The ACL tear group demonstrated a significantly lower mean NWI compared with the normal ACL group (0.2394 vs. 0.2637; p = 0.00001). Notch angle was also significantly smaller in patients with ACL tears (49.74° vs. 52.33°; p = 0.0056). A-shaped notch morphology showed a strong association with ACL rupture, whereas U-shaped and Ω-shaped notches were more frequently observed in knees with intact ACLs. Age did not show a significant association with ACL injury, while male predominance and right-sided involvement were more common in the ACL tear group.

Conclusion: Stenotic femoral intercondylar notch characteristics, including reduced NWI, smaller notch angle, and A-shaped configuration, are significantly associated with traumatic ACL injury. MRI-based morphometric evaluation may aid in identifying individuals at increased risk of ACL rupture and support targeted preventive strategies.

## Introduction

Anterior cruciate ligament (ACL) injury is a major cause of knee instability, particularly among young and physically active individuals [1,2]. Increasing participation in sports and recreational activities has contributed to a steady rise in the global incidence of ACL injuries [2]. Disruption of the ACL commonly results in pain, recurrent episodes of instability, and functional limitation, and often necessitates surgical reconstruction to restore joint stability and facilitate return to activity [3].

ACL injuries occur through both contact and non-contact mechanisms [4]. However, non-contact injuries are more frequent and are typically associated with sudden deceleration, pivoting, or twisting movements of the knee [4,5]. If inadequately managed, ACL deficiency may lead to persistent instability, secondary meniscal injury, and accelerated degenerative changes, thereby increasing the risk of early-onset osteoarthritis [6].

The femoral intercondylar notch houses the ACL and plays an important role in determining ligament biomechanics [7,8]. Variations in notch morphology, such as reduced notch width (NW), lower NW index (NWI), smaller notch angle, and specific notch configurations, have been proposed as intrinsic anatomical factors that may predispose individuals to ACL impingement and rupture [7,8]. Magnetic resonance imaging (MRI) enables accurate, non-invasive assessment of these morphometric parameters and is considered a reliable modality for anatomical evaluation of the knee.

Although several international studies have demonstrated a strong association between stenotic intercondylar notch morphology and ACL injury [7,8], evidence from Indian populations remains limited [9]. Given known anthropometric variations across different populations, region-specific data are essential to better characterize these anatomical risk factors [10]. Therefore, the present study aimed to evaluate MRI-based femoral intercondylar notch morphology and its association with traumatic ACL injury in a South Indian population.

## Materials and methods

Study design and setting

This prospective observational study was conducted at St. Peter’s Medical College, Hosur, Tamil Nadu, India, from June 2025 to January 2026 after approval from the Institutional Ethics Committee of St. Peter’s Medical College, Hosur, India (Approval No: SPMCH/IFC/AP/016/2025-2026). All MRI examinations were performed as part of routine clinical evaluation following knee trauma, and no additional imaging was obtained solely for research purposes.

Study population

A total of 108 patients aged 18-45 years who underwent knee MRI following acute traumatic injury were included. Traumatic mechanisms included road traffic accidents, sports-related injuries, accidental falls, and occupational trauma. Patients were divided into two equal groups: an ACL tear group (n = 54), comprising patients with partial or complete ACL disruption on MRI, and a normal ACL group (n = 54), comprising patients with traumatic knee symptoms but an intact ACL on imaging. In all cases, MRI was performed only on the symptomatic knee. Asymptomatic individuals and patients without a history of trauma were not included.

Exclusion criteria

Patients with a history of prior knee surgery, multi-ligament injuries, congenital knee abnormalities, degenerative joint disease, or other pathological conditions involving the ACL were excluded.

Sample size calculation

Sample size estimation was based on previously published data demonstrating a moderate association between femoral intercondylar NWI and ACL injury (correlation coefficient ≈ 0.26). This correlation was converted to an effect size using Cohen’s d. Assuming a two-tailed alpha level of 0.05, statistical power of 80%, and an effect size of 0.54, the minimum required sample size was calculated as 54 patients per group. Accordingly, a total of 108 patients were included.

MRI protocol

All MRI examinations were performed using a 1.5-Tesla MRI system. Imaging was conducted strictly as part of routine diagnostic care following knee trauma. For morphometric evaluation, axial T2-weighted fat-suppressed images were exclusively used to ensure uniformity and reproducibility.

Morphometric measurements

Morphometric evaluation of the femoral intercondylar notch was performed on standardized axial T2-weighted fat-suppressed MRI images. All measurements were obtained at the level of the popliteal groove. NW was defined as the minimum distance between the medial and lateral walls of the femoral intercondylar notch, and bicondylar width (BW) as the maximum transverse distance between the medial and lateral femoral condyles at the same axial level. The NW index (NWI) was calculated as the ratio of NW to BW (NW/BW).

To further characterize notch morphology, NW was measured at the popliteal groove level (NWp) and at the joint line level (NWj). The notch shape was classified as A-shaped when NWp < NWj, U-shaped when NWp = NWj, and Ω-shaped when NWp > NWj. The notch angle was measured by drawing reference lines along the medial and lateral margins of the intercondylar notch on axial images.

**Figure 1:**
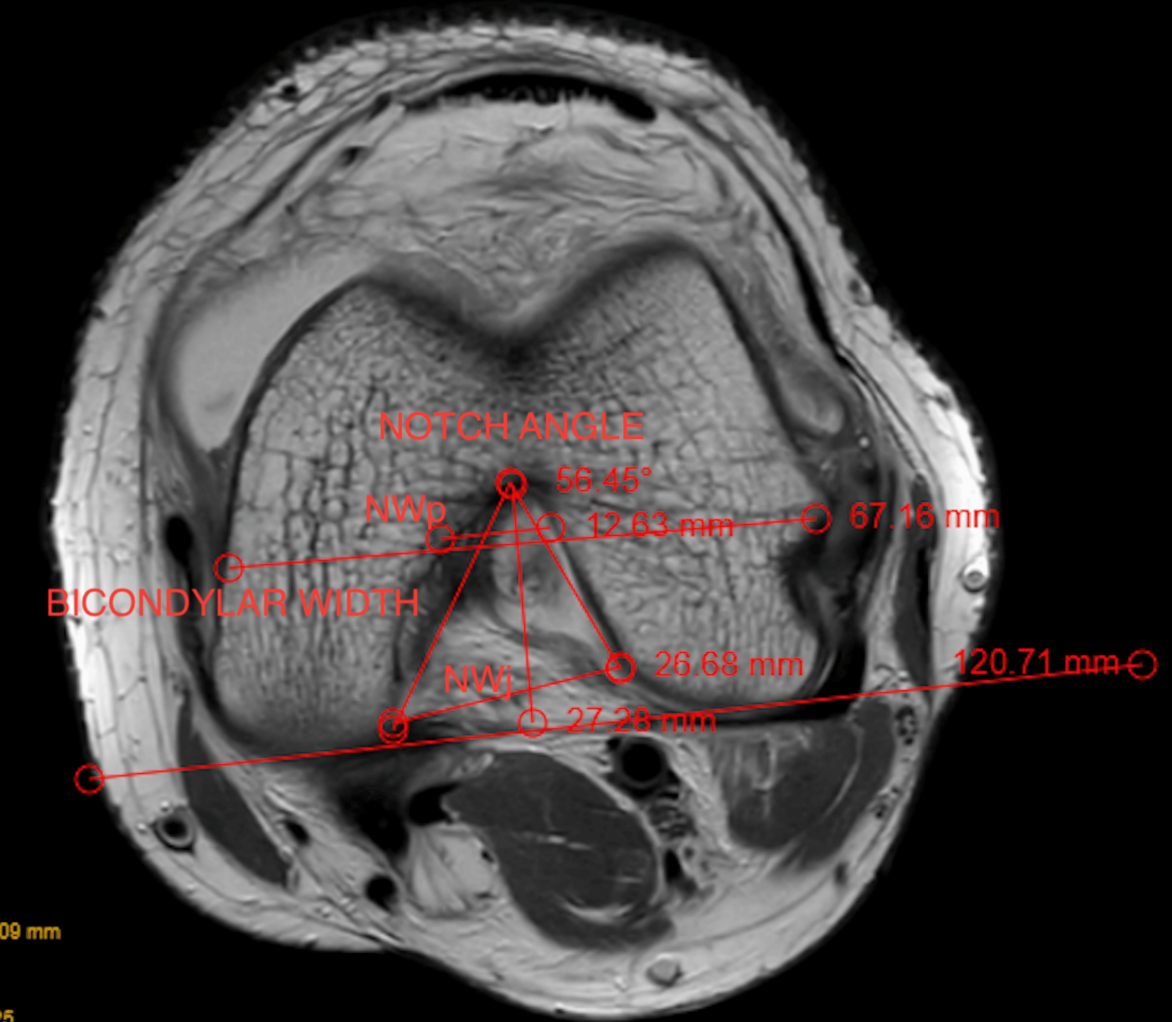
Axial T2-weighted MRI demonstrating femoral intercondylar notch morphometric measurements, including notch width (NW), bicondylar width (BW), NW index (NWI), notch angle, and notch shape classification Axial T2-weighted fat-suppressed magnetic resonance image of the knee obtained at the level of the popliteal groove, illustrating femoral intercondylar notch morphometric assessment. NW is measured as the minimum distance between the medial and lateral walls of the femoral intercondylar notch at the popliteal groove level. BW is measured as the maximum transverse distance between the medial and lateral femoral condyles on the same axial section. NWI is calculated as the ratio of NW to BW. The notch angle is measured by drawing reference lines along the medial and lateral margins of the intercondylar notch. Notch shape classification is based on the relationship between NW at the popliteal groove (NWp) and NW at the joint line (NWj): A-shaped when NWp < NWj, U-shaped when NWp = NWj, and Ω-shaped when NWp > NWj.

All measurements were performed using a standard DICOM workstation by a senior radiologist who was blinded to group allocation. Morphometric measurements and notch shape classification were performed on standardized axial T2-weighted fat-suppressed MRI images, as illustrated in Figure 1.

Statistical analysis

Statistical analysis was performed using IBM SPSS Statistics software, version 26 (IBM Corp., Armonk, NY, USA). Continuous variables were expressed as mean ± standard deviation and compared using the independent sample t-test. Categorical variables were analyzed using the chi-square (χ²) test. Data normality was assessed using the Shapiro-Wilk test. A p-value < 0.05 was considered statistically significant.

## Results

Demographic characteristics

The demographic characteristics of the study population are summarized in Table 1. The mean age of patients in the normal ACL group (31.37 ± 6.81 years) was comparable to that of the ACL tear group (31.26 ± 8.69 years), with no statistically significant difference observed (p = 0.94). Similarly, gender distribution did not differ significantly between the two groups (p = 0.21). Analysis of the side of involvement also revealed no significant association with ACL injury status (p = 0.66). These findings indicate that both groups were well matched with respect to baseline demographic variables, thereby minimizing potential confounding effects on the morphometric comparisons.

**Table 1:** Demographic characteristics of the study population Values are expressed as mean ± standard deviation for continuous variables and frequencies for categorical variables. Age was compared using the independent sample t-test, while gender and side of involvement were analyzed using the chi-square (χ²) test. ACL: anterior cruciate ligament

Variable	Normal ACL (n=54)	ACL Tear (n=54)	Test Statistic	p-value
Age (years, mean ± SD)	31.37 ± 6.81	31.26 ± 8.69	t = 0.07	0.94
Gender (male/female)	34 / 18	40 / 14	χ² = 1.57	0.21
Side (right/left)	27 / 27	29 / 24	χ² = 0.19	0.66

Morphometric parameters

**Figure 2:**
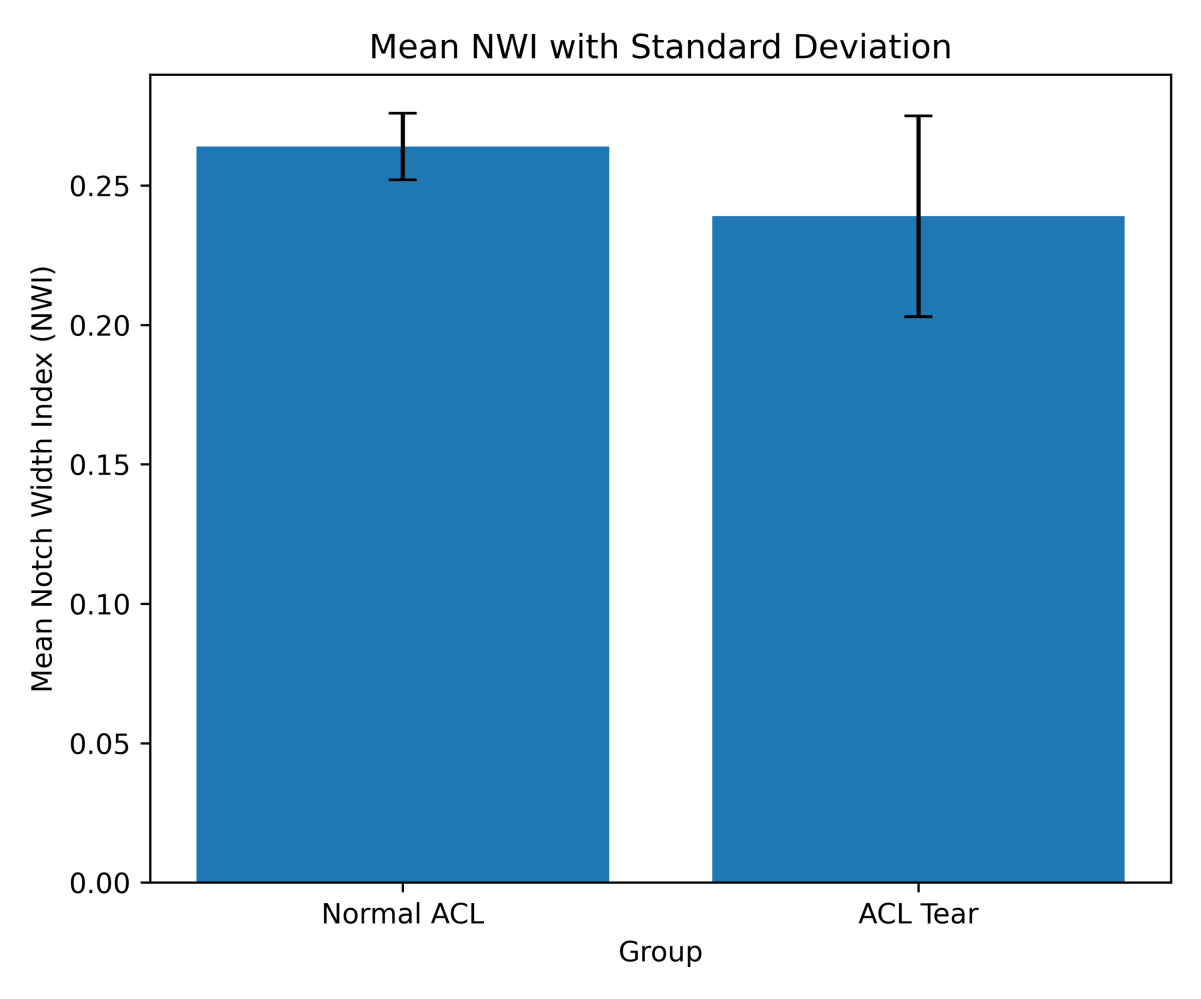
Bar chart showing mean notch width index (NWI) with standard deviation in normal anterior cruciate ligament (ACL) and ACL tear groups.

**Figure 3:**
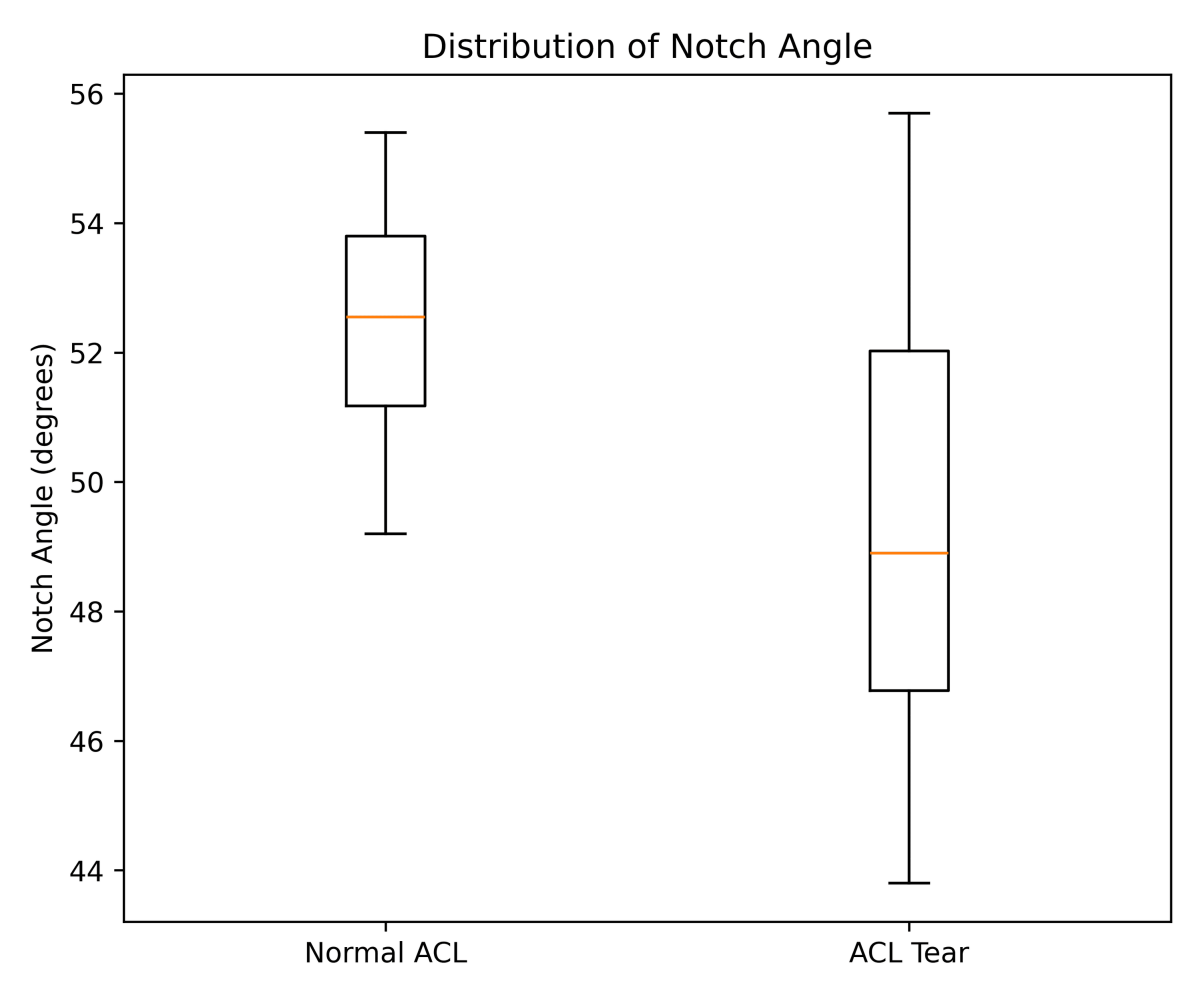
Box-and-whisker plot showing distribution of notch angle in normal anterior cruciate ligament (ACL) and ACL tear groups.

The comparison of morphometric parameters is illustrated in Figure 2 and Figure 3. The mean NWI was significantly lower in the ACL tear group (0.2394) compared with the normal ACL group (0.2637), indicating a relatively narrower intercondylar notch in patients with ACL injury (p = 0.00001) (Figure 2). This suggests that reduced NW may be an important intrinsic anatomical factor contributing to ACL vulnerability.

In addition, the mean notch angle was significantly smaller in the ACL tear group (49.74°) than in the normal ACL group (52.33°) (p = 0.0056) (Figure 3). A smaller notch angle reflects increased notch stenosis, which may predispose the ACL to mechanical impingement during knee motion, thereby increasing the likelihood of ligament rupture.

Notch shape distribution

The distribution of intercondylar notch shapes is presented in Table 2. A-shaped notch morphology was observed in 94% of ACL tear cases and demonstrated a very strong statistical association with ACL injury (χ² = 40.67, p = 1.47 × 10⁻⁹). In contrast, U-shaped notch morphology was predominantly observed in the normal ACL group, suggesting a comparatively lower risk of ACL injury in individuals with this configuration. Ω-shaped notches were relatively uncommon in both groups and did not show a meaningful pattern of association. These findings highlight notch shape as a particularly strong morphometric predictor of ACL injury.

**Table 2:** Distribution of intercondylar notch shapes in normal anterior cruciate ligament (ACL) and ACL tear groups. Values are expressed as frequencies. Statistical analysis was performed using the chi-square (χ²) test to assess the association between notch shape and ACL injury status.

Notch Shape	Normal ACL	ACL Tear	χ²	p-value
A-shaped	20	51	40.67	1.47×10⁻⁹
U-shaped	28	1		
Ω-shaped	6	2		

## Discussion

The present study demonstrates a significant association between femoral intercondylar notch morphology and traumatic ACL injury. Among the evaluated parameters, reduced NWI emerged as the strongest morphometric predictor of ACL rupture, supporting the hypothesis that a stenotic intercondylar notch increases the risk of mechanical impingement on the ligament during knee motion [7,8,10]. A narrower notch may reduce the available space for the ACL, thereby predisposing it to repetitive microtrauma and eventual failure, particularly during high-demand activities involving pivoting and sudden directional changes.

The findings of this study are in agreement with previously published literature. The systematic review and meta-analysis by Andrade et al. identified reduced NW and lower NWI as important intrinsic anatomical risk factors for ACL injury [1]. Similarly, Akgün and Tekcan reported significantly lower NWI and smaller notch angles in ACL-injured patients on MRI, further reinforcing the role of notch stenosis in ligament vulnerability [7]. These consistent observations across multiple populations suggest that intercondylar notch morphology represents a reliable and reproducible anatomical parameter associated with ACL injury risk.

Yellin et al. demonstrated comparable associations in pediatric populations, indicating that notch morphology may reflect congenital or developmental characteristics that persist into adulthood and influence injury susceptibility over time [8]. Indian studies by Patidar et al. and Fahim et al. also reported a strong correlation between A-shaped notch configuration, reduced NWI, and ACL tears, highlighting the relevance of these anatomical risk factors in regional populations [9,10]. The high prevalence of A-shaped notch morphology among ACL-injured patients in the present study further supports its role as a significant morphometric indicator of injury risk.

Although a higher proportion of male patients was observed in the ACL tear group, gender was not found to be a statistically significant factor in the present analysis. This trend may reflect greater exposure to high-risk physical activities, occupational demands, or sports participation among males rather than inherent anatomical differences [2,11]. These findings emphasize the importance of considering both anatomical and behavioral factors when evaluating ACL injury risk.

From a clinical perspective, MRI-based assessment of femoral notch morphology provides a practical, non-invasive method for identifying individuals who may be predisposed to ACL injury. It is important to note that all MRI examinations in this study were performed strictly as part of routine clinical evaluation following knee trauma, and no asymptomatic individuals were subjected to imaging solely for research purposes, as detailed in the Methods section. Early recognition of high-risk anatomical features may assist clinicians in counseling patients, implementing targeted preventive training programs, and planning surgical strategies, particularly in athletes and physically active individuals.

Despite the significant findings, the present study has certain limitations that should be acknowledged. This was a single-center study with a relatively modest sample size, which may limit the generalizability of the results to broader populations. Only MRI-based morphometric parameters were evaluated, and functional or biomechanical factors such as activity level, limb alignment, and neuromuscular control were not assessed, which could also influence ACL injury risk. Additionally, although measurements were performed by an experienced radiologist, inter-observer variability was not analyzed. Future multicenter studies with larger sample sizes and inclusion of additional clinical and biomechanical variables may provide a more comprehensive understanding of the relationship between femoral notch morphology and ACL injury.

## Conclusions

This study demonstrates that femoral intercondylar notch morphology plays an important role in traumatic ACL injury, with stenotic notch features showing a strong association with ACL tears. Reduced NWI, smaller notch angle, and A-shaped notch configuration were identified as key anatomical characteristics linked to increased injury risk. These observations emphasize the value of MRI-based morphometric assessment as a simple and non-invasive approach for identifying individuals who may be at higher risk of ACL rupture. Incorporating notch morphology evaluation into routine clinical practice may support early risk stratification, improve patient counseling, and facilitate the development of targeted preventive strategies in physically active populations.
